# Na,K-ATPase β_1_-subunit is a target of sonic hedgehog signaling and enhances medulloblastoma tumorigenicity

**DOI:** 10.1186/s12943-015-0430-1

**Published:** 2015-08-19

**Authors:** Seung Joon Lee, Alisa Litan, Zhiqin Li, Bruce Graves, Stephan Lindsey, Sonali P. Barwe, Sigrid A. Langhans

**Affiliations:** Nemours/Alfred I. duPont Hospital for Children, Rockland Center I, 1701 Rockland Road, Wilmington, DE 19803 USA

**Keywords:** Medulloblastoma, Na,K-ATPase, Sonic hedgehog, Cerebellar granule cell, Brain tumor

## Abstract

**Background:**

The Sonic hedgehog (Shh) signaling pathway plays an important role in cerebellar development, and mutations leading to hyperactive Shh signaling have been associated with certain forms of medulloblastoma, a common form of pediatric brain cancer. While the fundamentals of this pathway are known, the molecular targets contributing to Shh-mediated proliferation and transformation are still poorly understood. Na,K-ATPase is a ubiquitous enzyme that maintains intracellular ion homeostasis and functions as a signaling scaffold and a cell adhesion molecule. Changes in Na,K-ATPase function and subunit expression have been reported in several cancers and loss of the β_1_-subunit has been associated with a poorly differentiated phenotype in carcinoma but its role in medulloblastoma progression is not known.

**Methods:**

Human medulloblastoma cell lines and primary cultures of cerebellar granule cell precursors (CGP) were used to determine whether Shh regulates Na,K-ATPase expression. Smo/Smo medulloblastoma were used to assess the Na,K-ATPase levels *in vivo*. Na,K-ATPase β_1_-subunit was knocked down in DAOY cells to test its role in medulloblastoma cell proliferation and tumorigenicity.

**Results:**

Na,K-ATPase β_1_-subunit levels increased with differentiation in normal CGP cells. Activation of Shh signaling resulted in reduced β_1_-subunit mRNA and protein levels and was mimicked by overexpression of Gli1and Bmi1, both members of the Shh signaling cascade; overexpression of Bmi1 reduced β_1_-subunit promoter activity. In human medulloblastoma cells, low β_1_-subunit levels were associated with increased cell proliferation and *in vivo* tumorigenesis.

**Conclusions:**

Na,K-ATPase β_1_-subunit is a target of the Shh signaling pathway and loss of β_1_-subunit expression may contribute to tumor development and progression not only in carcinoma but also in medulloblastoma, a tumor of neuronal origin.

## Background

The Sonic hedgehog (Shh) signaling pathway is critical for patterning, proliferation, and differentiation in embryonic development. Aberrant activation of Shh signaling has been associated with tumorigenesis in various malignancies [1] including medulloblastoma, a pediatric brain cancer of the cerebellum [2]. Medulloblastoma is divided into four biologically different subsets of tumors defined as Wnt, Shh, Group 3 and Group 4 that have distinct genetic profiles, pathway signatures, and clinicopathological properties [3, 4]. Aberrant activation of Shh signaling has been implicated in about one third of medulloblastoma [5, 6]. Tumors of this group are thought to originate from cerebellar granule cell precursor (CGP) cells that are located in the external granule layer (EGL) of the cerebellum, a germinal zone harboring actively proliferating progenitor cells [7, 8]. A tightly regulated switch to the post-mitotic stage correlates with differentiation and inward migration of the granule neurons to form the internal granule layer (IGL). Disruption of the spatio-temporal dynamics of CGP proliferation in the EGL, cell cycle exit, formation of parallel fibers and migration into the IGL are thought to play a role in medulloblastoma development [7, 8]. However, factors that regulate these dynamics are poorly characterized.

Shh is a secreted protein released from Purkinje cells, being responsible for cerebellar patterning, polarity and development [2] and is a potent mitogen for CGPs [9]. In general, the Shh pathway is inactive in the absence of ligand. The transmembrane receptor Patched 1 (Ptch1) inhibits the activity of the Smoothened (Smo) receptor. The transcription factor Gli is prevented from entering the nucleus through interactions with cytoplasmic proteins like Suppressor of fused homolog (Sufu) and transcriptional activation of Shh target genes is repressed [1]. Activation of the pathway is initiated through Shh ligand binding to Ptch1 resulting in de-repression of Smo. This activates a cascade leading to the translocation of the active form of Gli to the nucleus to activate gene expression. In medulloblastoma, activating mutations in three Shh pathway components, Ptch1, Smo, and Sufu, have been identified [10, 11]. Target genes of the Shh pathway include polycomb group proteins that are transcription regulatory proteins that form protein complexes to repress gene transcription [12]. The polycomb protein Bmi1 acts downstream of the Shh pathway to control CGP proliferation [13]. Recent studies showed that Bmi1 is aberrantly expressed in medulloblastoma which correlates with the activation of the Shh pathway [13] and is required for Shh driven medulloblastoma expansion [14, 15]. The target genes of Bmi1 in medulloblastoma are not well-known.

Na,K-ATPase is a ubiquitous membrane protein that maintains intracellular ion homeostasis and is critical to the normal function of higher eukaryotic cells, including neurons. The enzyme consists of a catalytic α-subunit and a β-subunit and pumps sodium ions out and potassium ions into the cell at the expense of ATP thereby generating a sodium gradient across the plasma membrane [16, 17]. In recent years, studies from our laboratory and other groups have established additional roles for Na,K-ATPase as a signaling scaffold and a cell adhesion molecule [18–23]. Signaling pathways modulated by Na,K-ATPase have been linked to cancer development and progression including cell growth, cell adhesion, and cell motility. A cell adhesion function has been ascribed to both the β_1_ and β_2_ isoforms. Indeed, the β_2_-subunit was initially described as adhesion molecule on glia (AMOG) and mediates neuron-astrocyte adhesion and neural cell migration [24]. A recent study suggested that increasing loss of AMOG plays a role in the invasion pattern in the malignant progression of gliomas [25–27]. We have shown that loss of β_1_-subunit expression is associated with a poorly differentiated phenotype found in carcinoma cells [21, 28–30]. Changes in Na,K-ATPase function and expression have been reported in various cancers [31] and may occur at very early stages of tumorigenesis [32, 33], suggesting that altered Na,K-ATPase subunit expression and function may contribute to tumor development and progression. We previously found that the β_1_-subunit is repressed by the transcription factor Snail [29] and activation of transforming growth factor (TGF)-β signaling resulted in reduced β_1_-subunit expression in epithelial cells [34, 35]. Whether activation of Shh signaling regulates β_1_-subunit levels is not known.

In this study, we analyzed the expression of Na,K-ATPase subunits in medulloblastoma tumors of Smo/Smo mice, a transgenic medulloblastoma mouse model with aberrant activation of Shh signaling [36]. The levels of the β_1_-subunit of Na,K-ATPase were drastically reduced in medulloblastoma tumors when compared to cerebellum of wild-type mice and activation of Shh signaling repressed the β_1_-subunit expression in differentiating primary CGP cultures. Overexpression of Gli1 and Bmi1, both downstream effectors of Shh signaling, resulted in reduced β_1_-subunit mRNA and protein expression. Furthermore, shRNA-mediated knockdown of the β_1_-subunit in a human medulloblastoma cell line increased cell proliferation and tumorigenicity in xenografts in immunocompromised mice suggesting that loss of β_1_-subunit may contribute to medulloblastoma progression.

## Results

### Reduced expression of Na,K-ATPase β_1_-subunit in medulloblastoma tumors

**Fig. 1:**
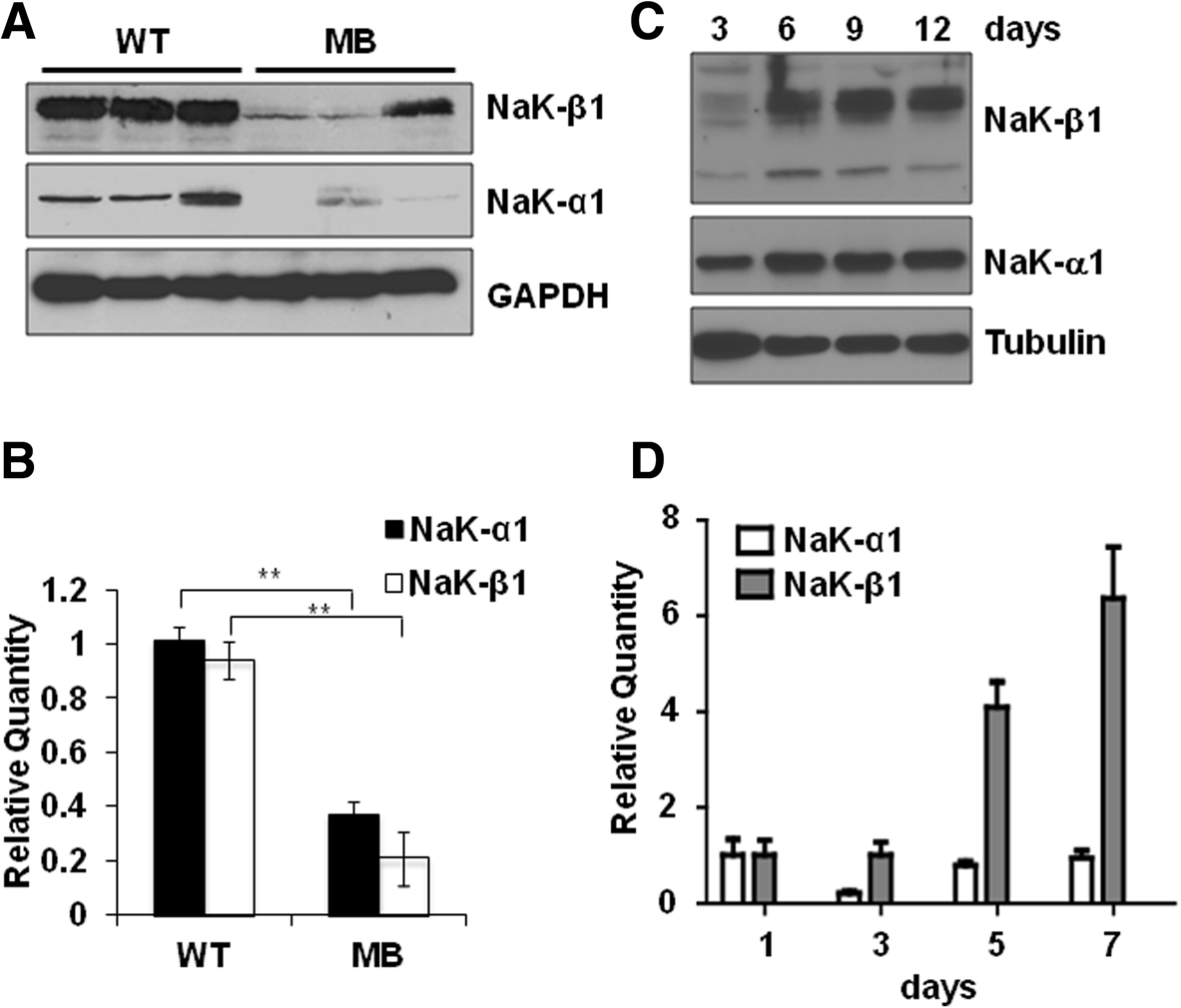
Na,K-ATPase subunits in medulloblastoma and CGP cells. **a**. Na,K-ATPase α_1_- and β_1_-subunit expression in cerebellum from 6 month old WT C57BL6/J mice (WT) and tumors from age-matched Smo/Smo mice. An immunoblot for GAPDH confirmed equal loading of protein. **b**. Na,K-ATPase α_1_-subunit and β_1_-subunit mRNA levels in WT and medulloblastoma cerebellum normalized to beta-2 microglobulin. For both α_1_- and β_1_-subunit the difference between WT and medulloblastoma cerebellum is statistically significant (*p* < 0.005) (*n* = 3). **c**. Primary CGP cells isolated from C57BL/6 J mice were cultured in vitro for 3, 6, 9 and 12 days and then lysed for immunoblotting with indicated antibodies. α-tubulin was used as a loading control. **d**. mRNA levels of α_1_- and β_1_-subunit during differentiation of CGP cells cultured *in vitro* and normalized to beta-2 microglobulin

We previously found that the Na,K-ATPase β_1_-subunit expression correlated with differentiation, being high in well differentiated epithelial cells and low in poorly differentiated cancer cells [21, 28–30]. However, it is not known whether a similar correlation exists in neuronal tumors. Since normal cerebellar granule cells express high levels of the β_1_ isoform [37], we first compared the β_1_-subunit levels in normal cerebella and medulloblastoma tumors from Smo/Smo mice. Smo/Smo mice are a transgenic medulloblastoma mouse model with expression of a constitutively activated form of the *Smo* gene in CGP cells and form medulloblastoma tumors at a high incidence and early onset [36]. Immunoblotting revealed drastically reduced β_1_-subunit levels in medulloblastoma tumors as compared to normal cerebellum of age-matched wildtype C57BL/6 mice (Fig. 1a). Consistent with the reduced β_1_-subunit expression, β_1_-subunit mRNA levels in tumors were only about 20 % of the levels of normal cerebellum (Fig. 1b). Protein and mRNA levels of the α_1_-subunit, the major isoform in CGP cells [37] were also reduced. Furthermore, the β_1_-subunit protein (Fig. 1c) and mRNA (Fig. 1d) levels increased with time in differentiating primary cultures of CGP cells isolated from normal cerebella suggesting that the β_1_-subunit levels increase with the differentiation of CGP cells.

### Reduced Na,K-ATPase β_1_-subunit expression increases cell proliferation and tumorigenicity

**Fig. 2:**
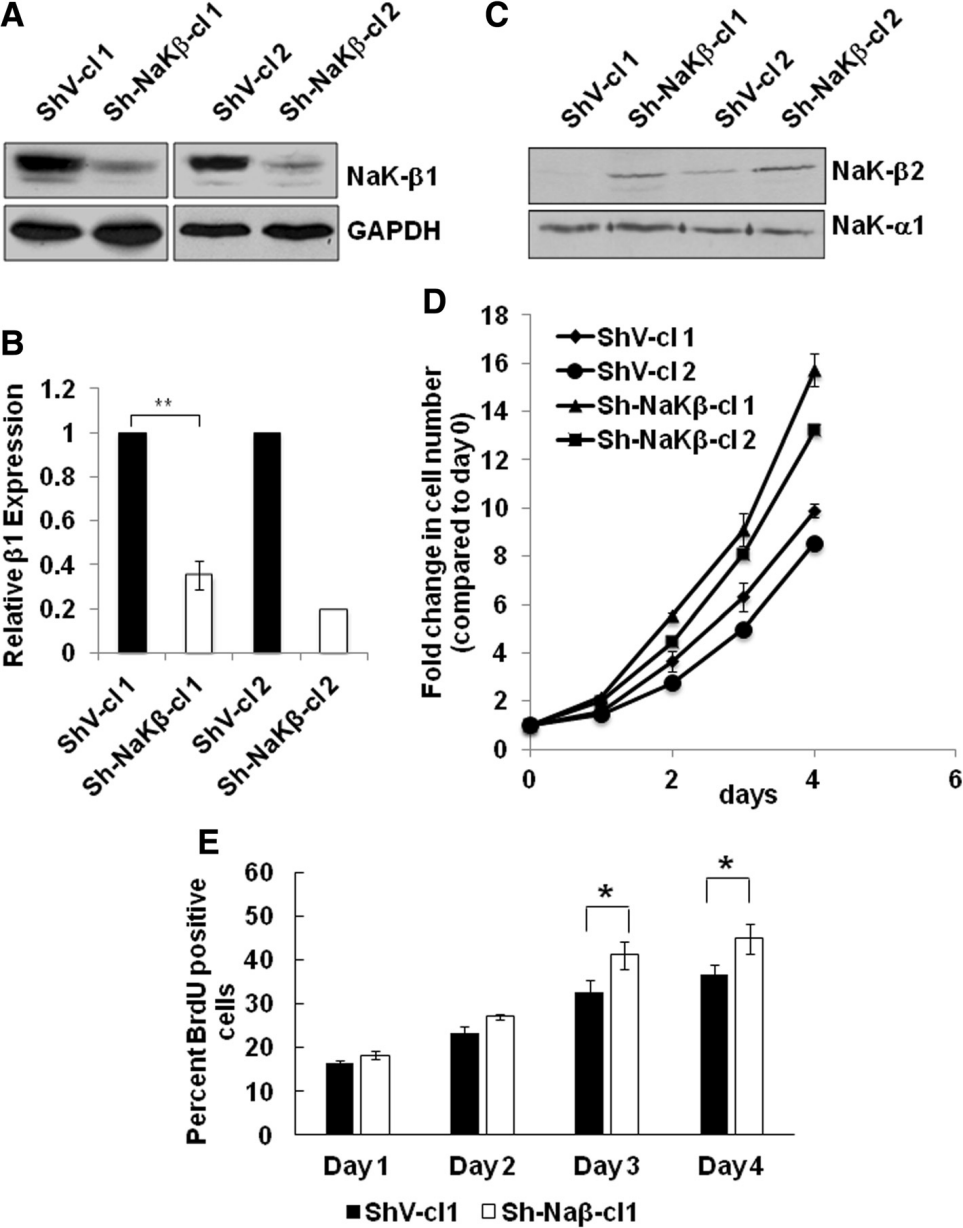
Knockdown of Na,K-ATPase β_1_-subunit in medulloblastoma cells increases cell proliferation. **a**. Na,K-ATPase β_1_-subunit protein levels in two independent clones of shRNA-mediated β_1_-subunit knockdown in DAOY cells (Sh-NaKβ-cl1 and cl2). ShV-cl1 and cl2 are respective scrambled shRNA transfected control clones that were obtained in parallel with each of the two independent selections. **b**. Quantification of β_1_-subunit levels in knockdown cells relative to the levels in the respective control clones from three (Sh-NaKβ-cl1 and ShV-cl1; *p* < 0.005) or two (Sh-NaKβ-cl2 and ShV-cl2) immunoblots. **c**. Na,K-ATPase β_2_-isoform and α_1_-subunit expression in β_1_-subunit knockdown cells. **d**. Cell proliferation of β_1_-subunit knockdown clones expressed as fold change relative to cell number at day 0. The differences in fold change between ShV-cl 1 and Sh-NaKβ-cl 1 were statistically significant at days 2, 3, and 4 (*p* < 0.05). **e**. BrdU uptake in β_1_-subunit knockdown cells. There was a significant difference in percent BrdU positive cells between cell lines (*p* < 0.005, two way ANOVA). The difference in percent BrdU positive cells was significant starting on day 3 (* = *p* < 0.05)

To test whether loss of β_1_-subunit expression affects medulloblastoma progression, we used an RNA interference approach to knockdown β_1_-subunit in the human medulloblastoma cell line DAOY. From two independent transfections and selections we obtained two clones of β_1_-subunit knockdown cells with a 65 % (Sh-NaKβ-cl1) and 81 % (Sh-NaKβ-cl2) reduction in β_1_-subunit expression compared to the respective control cells (ShV-cl1 and ShV-cl2) that were transfected and selected in parallel with each clone (Fig. 2a, b). The α_1_-subunit levels were comparable in control and knockdown cells, which is likely due to the compensatory increase of the β_2_-isoform in knockdown cells (Fig. 2c). Interestingly, cells from both β_1_-subunit knockdown clones proliferated 1.6 +/− 0.05 (cl1) and 1.5 (cl2) times faster than the respective control cells by day 4 (Fig. 2d). The increase in proliferation in β_1_-subunit knockdown cells was further confirmed by BrdU uptake experiments (Fig. 2e).

**Fig. 3:**
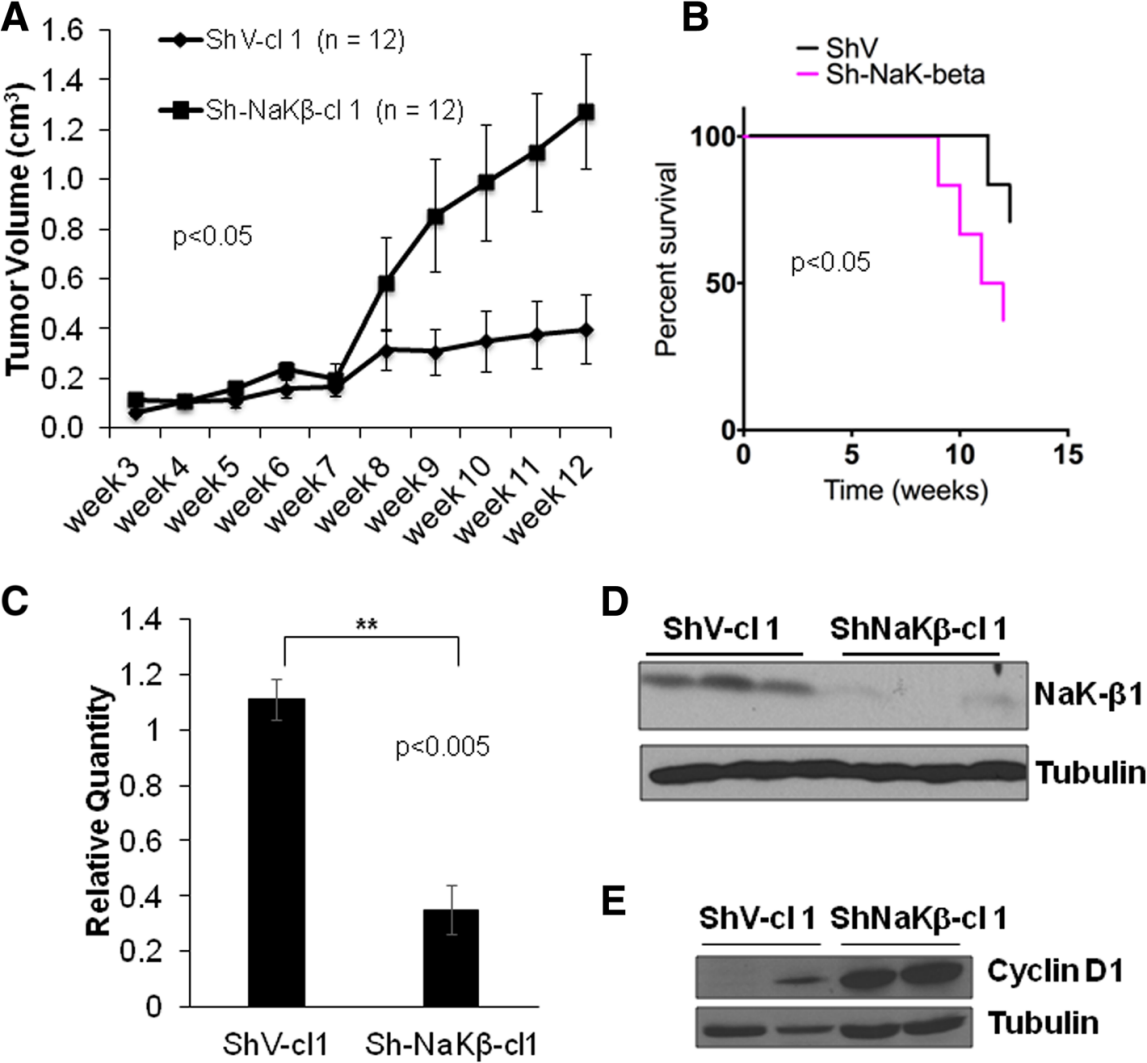
Tumor xenografts of Na,K-ATPase β_1_-subunit knockdown cells. **a**. Tumor volume of subcutaneous xenograft tumors injected into immunocompromised mice (*n* = 12). The difference in the overall mean volume between the group of ShV-cl1 and Sh-NaKβ-cl1 tumors was significant (*p* < 0.05, determined using a mixed effect model with AR(1) correlation structure). **b**. Kaplan-Meier survival curve of mice with ShV-cl1 (ShV) and Sh-NaKβ-cl1 (Sh-NaK-beta) tumors (*p* < 0.05). **c**. Average β_1_-subunit mRNA levels from three representative tumors of ShV-cl1 and Sh-NaKβ-cl1 cells normalized to beta-2 microglobulin. The difference in mRNA expression was significant (** = *p* < 0.005). **d**. β_1_-subunit protein levels in xenograft tumors shown in **c**. An immunoblot for tubulin served as loading control. **e**. Cyclin D1 levels in xenograft tumors shown in **c**. An immunoblot for tubulin served as loading control

Furthermore, in subcutaneous tumor xenografts in immunocompromised mice there was a significant difference in the overall mean volume between the group of vector control (ShV-cl1) and β_1_-subunit knockdown (Sh-NaKβ-cl1) (Fig. 3a). The overall mean volume (SE) was 0.236 (0.112) cm^3^ and 0.563 (0.112) cm^3^ in ShV-cl1 and Sh-NaKβ-cl1, respectively. The difference in the mean (SE) was 0.327 (0.158) cm^3^, *p* = 0.049. The changes between the groups over time were significantly different (*p*-value (for Time*Group) <0.001) (*n* = 12). Week-wise, the mean differences between two groups were highly significant after week 8. A survival analysis estimated the risk of dying in Sh-NaKβ-cl1 as 4.8 times higher compared to the risk of dying in ShV-cl1 and a Kaplan Meier survival function was used to compare the probability of surviving between two groups (Fig. 3b). qPCR (Fig. 3c) and immunoblot (Fig. 3d) analysis from tumors obtained at termination of the study at 12 weeks, confirmed that the β_1_-subunit knockdown had been maintained *in vivo*. In addition, tumors from β_1_-subunit knockdown cells had higher cyclin D1 levels (Fig. 3e). At the same time, cyclin D1 expression itself did not result in reduced β_1_-subunit levels (data not shown). Thus, reduced β_1_-subunit expression in medulloblastoma cells is associated with increased proliferation and tumorigenicity, suggesting that loss of the β_1_-subunit may contribute to medulloblastoma progression rather than being a byproduct of increased proliferation.

### Activation of Shh signaling suppresses the Na,K-ATPase β_1_-subunit

**Fig. 4:**
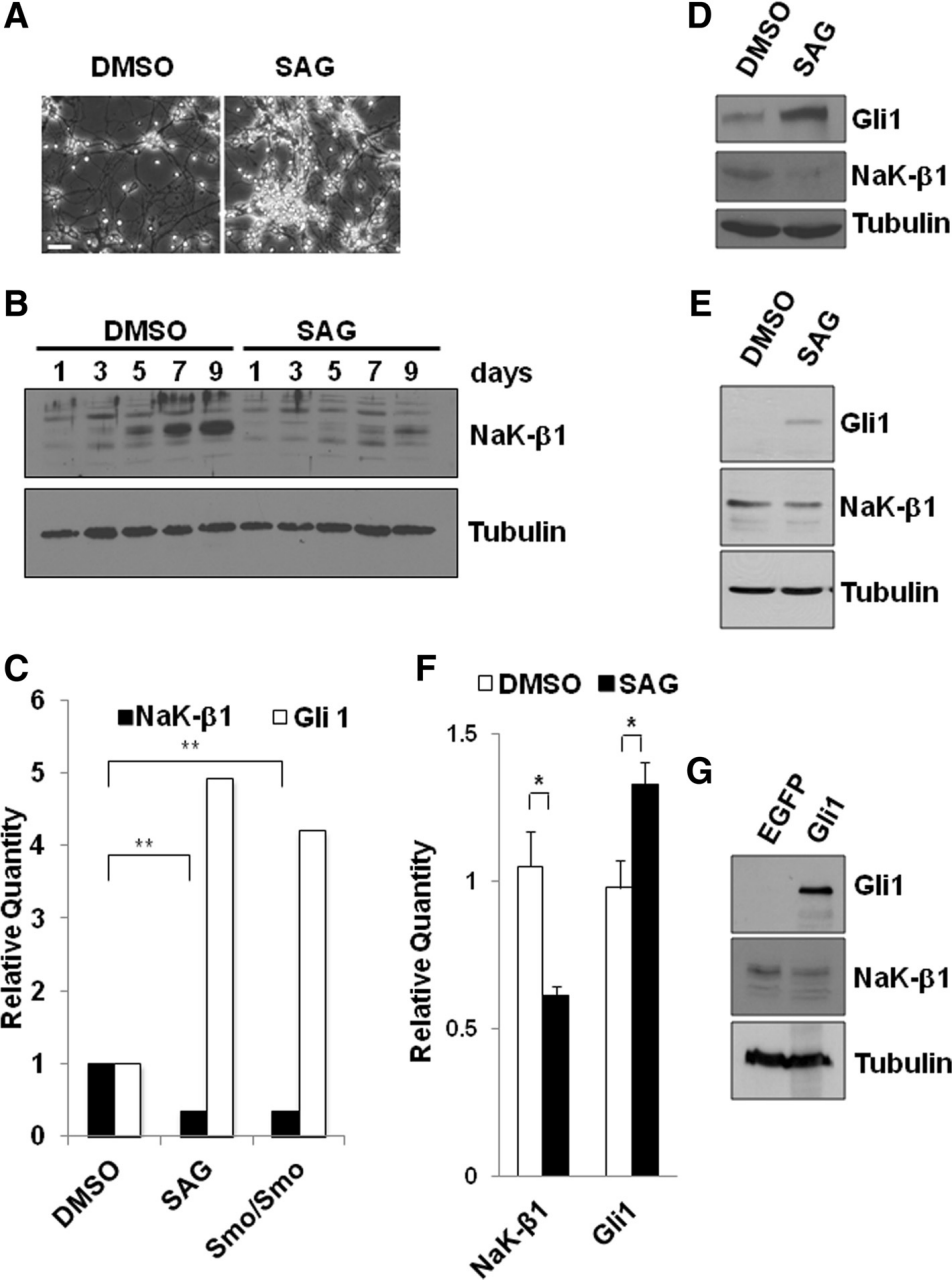
Activation of Shh signaling prevents Na,K-ATPase β_1_-subunit upregulation during CGP differentiation. **a**. Phase contrast images showing proliferation of CGP cells after six days of treatment with 0.1 μM SAG. Scale bar, 100 μm. **b**. *In vitro* cultured CGP cells were incubated with either DMSO or 0.1 μM SAG for the indicated period of time and β_1_-subunit levels were determined by immunoblotting. α-tubulin was used to ensure equal loading. **c**. β_1_-subunit mRNA levels in CGP cells cultured from WT mice. mRNA levels are from CGP cells treated with DMSO or 0.1 μM SAG for seven days, and CGP cells cultured from Smo/Smo mice. The mRNA levels were normalized to control cells treated with DMSO. The differences in β_1_-subunit mRNA levels between DMSO and SAG treated and DMSO and Smo/Smo CGP cells were significant (** = *p* < 0.005). **d**. Immunoblots for β_1_-subunit and Gli1 in ONS-76 cells treated for 48 h with 0.1 μM SAG. α-tubulin served as loading control. **e**. Immunoblots for β_1_-subunit and Gli1 in DAOY cells treated for 48 h with 0.1 μM SAG. α-tubulin served as loading control. The β_1_-subunit level relative to the level of α-tubulin in SAG-treated cells was reduced to 60 % of the β_1_-subunit level in DMSO-treated control cells. **f**. β_1_-subunit and Gli1 mRNA levels in DAOY cells treated for 2 h with 0.1 μM SAG. β-actin was used as the endogenous control. The difference between the mRNA levels of β_1_-subunit and Gli1 was significant (* = *p* < 0.05). **g**. β_1_-subunit levels in Gli1 overexpressing cells. 293 T cells were transfected with pEGFPC1-Gli1 or pEGFPC1 vector as control for 72 h. Immunoblots for β_1_-subunit and Gli1 are shown. α-tubulin served as loading control. The β_1_-subunit level relative to the level of α-tubulin in Gli-transfected cells was only 70 % of the β_1_-subunit level in vector only-transfected cells

Since Shh signaling is a major pathway affected in medulloblastoma and Smo/Smo mice express constitutively active Smo, we next tested whether activation of Shh affects the β_1_-subunit levels. CGP cells isolated from normal cerebellum were differentiated in culture or treated with the Smo agonist SAG to induce cell proliferation (Fig. 4a). Indeed, SAG treatment prevented the increase in β_1_-subunit expression (Fig. 4b) that was found in control CGP cells (Figs. 1c, 4b). Similar to Smo/Smo tumors, β_1_-subunit downregulation upon Shh activation occurred at the transcriptional level as SAG treatment not only increased Gli1 levels but also reduced β_1_-subunit mRNA levels to 34 % of that of control cells (Fig. 4c). Interestingly, the β_1_-subunit mRNA level in SAG-treated cells was similar to that of primary cultures of tumor cells from Smo/Smo mice (Fig. 4c). Furthermore, in ONS-76 and DAOY cells SAG-treatment reduced the β_1_-subunit levels by about 40 % as compared to control cells and clearly decreased mRNA levels (Fig. 4d-f). However, compared to primary CGP cultures, SAG-treatment of DAOY cells did not reduce the β_1_-subunit as drastically. Probably these cells were less sensitive to SAG-treatment which is consistent with the lower levels of Gli1 observed in these cells. To further investigate whether activation of Shh signaling results in reduced β_1_-subunit levels, we transiently transfected HEK293T cells with EGFP-tagged Gli1. As compared to EGFP vector control cells, the β_1_-subunit in Gli1 overexpressing cells was reduced by 30 % (Fig. 4g). Thus, activation of Shh signaling represses β_1_-subunit mRNA levels and protein expression.

### Bmi1 represses β_1_-subunit expression

**Fig. 5:**
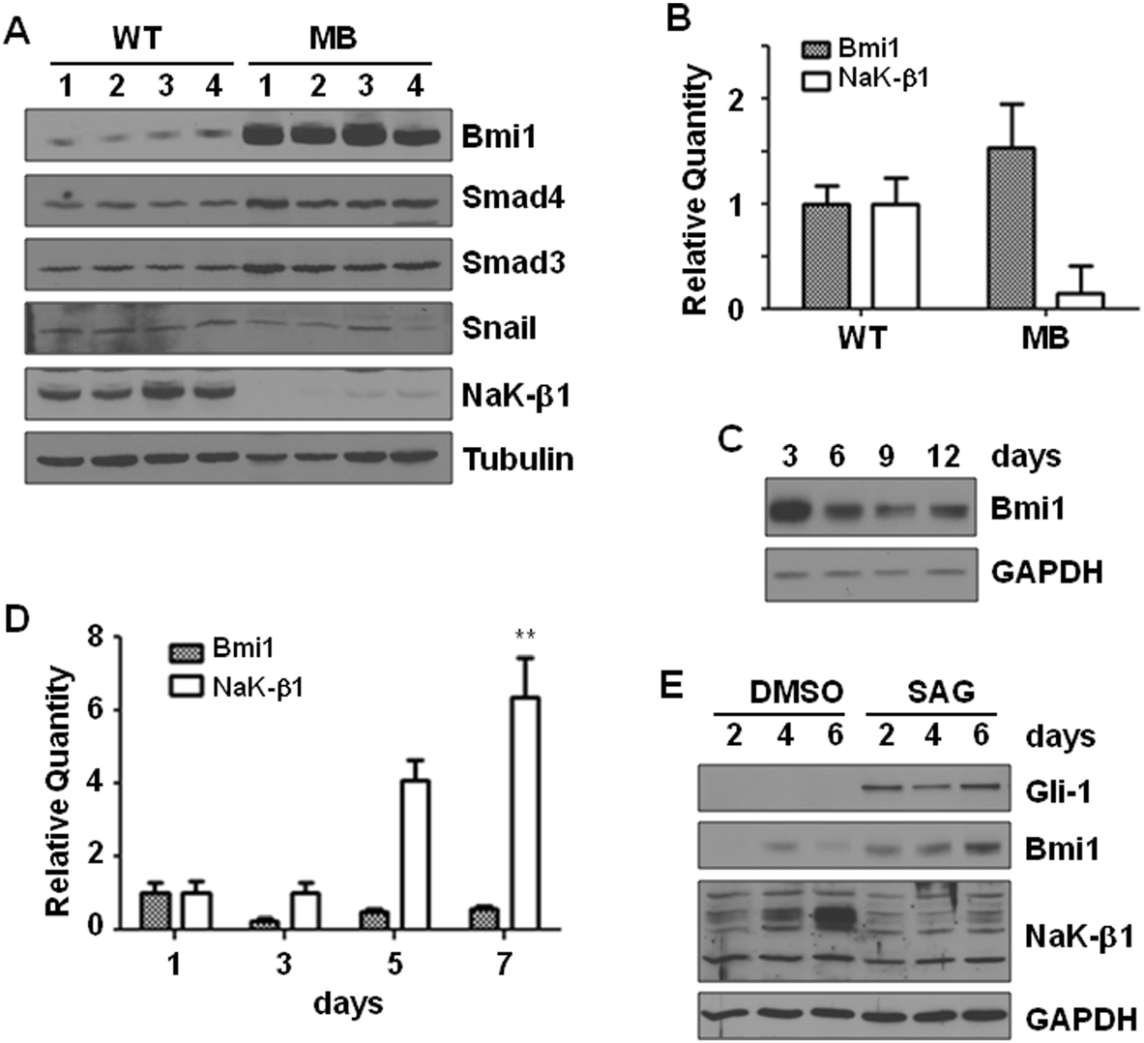
Inverse relationship between Bmi1 and Na,K-ATPase β_1_-subunit levels in medulloblastoma and CGP cells. **a**. Protein extracts obtained from four Smo/Smo medulloblastoma and cerebellum of four wild type mice were immunoblotted with the indicated antibodies. α-tubulin was used as loading control. **b**. mRNA levels of Bmi1 and β_1_-subunit in medulloblastoma and cerebellum as determined by qRT-PCR. **c**. Protein levels of Bmi1 in differentiating CGP cells. GAPDH was used to ensure equal loading. **d**. mRNA levels of Bmi1 and β_1_-subunit in CGP cells differentiating *in vitro*. The difference in β_1_-subunit mRNA expression was significant during CGP differentiation (** = *p* < 0.001, one way ANOVA), while the difference in Bmi1 expression was not significant (one way ANOVA). **e**. CGP cells grown *in vitro* were exposed to either DMSO or 0.1 μM SAG for indicated time points and the lysates were immunoblotted with the indicated antibodies. GAPDH served as loading control

**Fig. 6:**
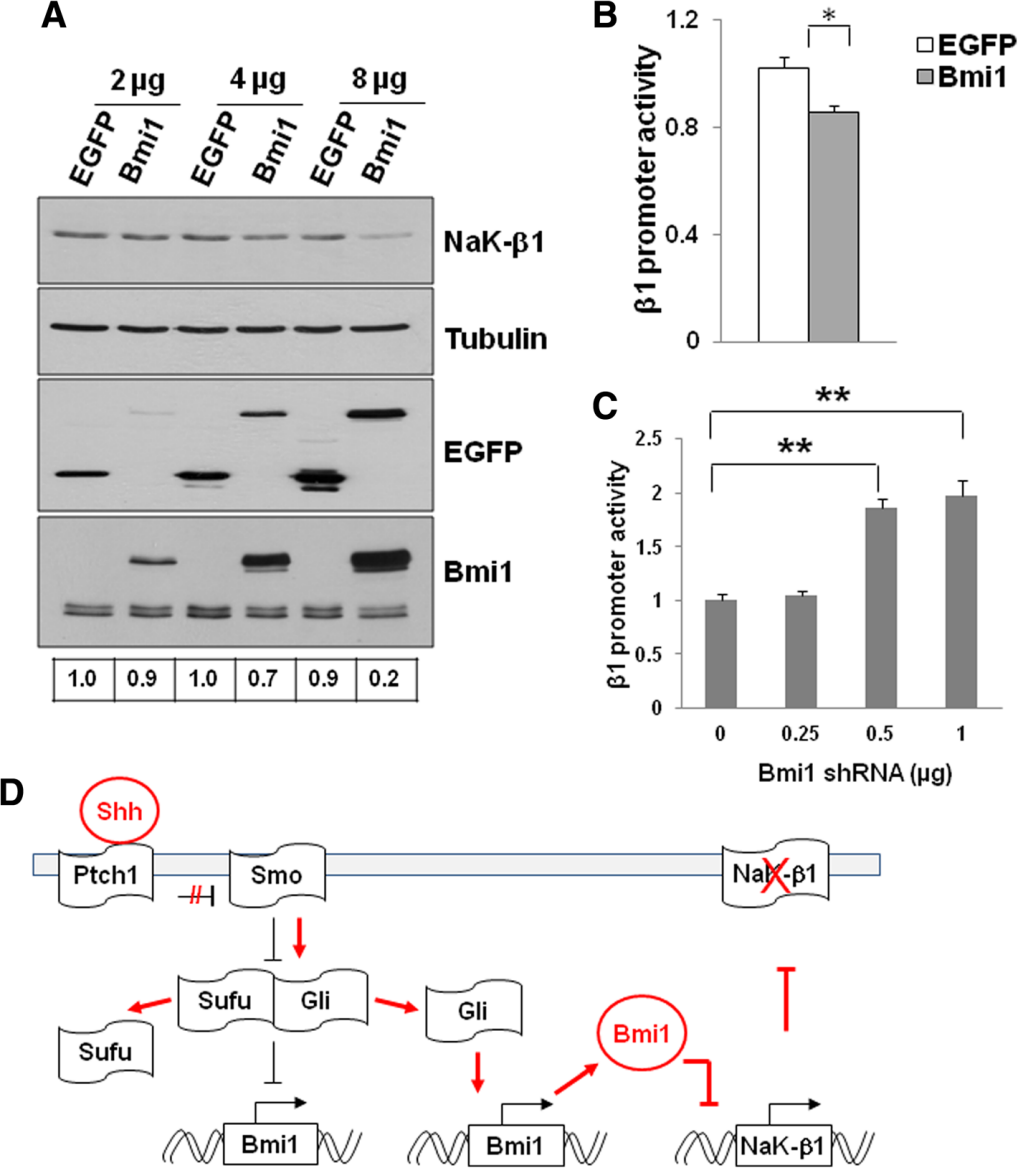
Bmi1 represses Na,K-ATPase β_1_-subunit. **a**. β_1_-subunit expression in 293 T cells transiently transfected with increasing amounts of pEGFPC1 or pEGFPC1-Bmi1. Immunoblots for β_1_-subunit, GFP, and Bmi1 are shown. α-tubulin serves as the loading control. **b**. Bmi1 decreased β_1_-subunit promoter activity in 293 T cells transfected with pEGFPC1 or pEGFPC1-Bmi1 together with β_1_-subunit promoter-Luc and Renilla control vector. 48 h after transfection, dual-luciferase assays were performed and Firefly luciferase activity was normalized to the Renilla luciferase signal to control for transfection efficiency. Data shown are from three experiments (* = *p* < 0.05). **c**. Knockdown of Bmi1 increased β_1_-subunit promoter activity in 293 T cells. 293 T cells were co-transfected with empty vector or human Bmi1 shRNA construct together with β_1_-subunit promoter-Luc and Renilla control vector. 48 h after transfection, dual-luciferase assays were performed as in **b**. Data shown are representative of two independent experiments (** = *p* < 0.01) **d**. Schematic model depicting Shh-mediated repression of Na,K-ATPase β_1_-subunit resulting in increased proliferation and tumorigenicity

Gli proteins can function as transcriptional activators and repressors, but Gli1 is mostly known as a transcriptional activator. One of the target genes of Gli1 is the polycomb protein Bmi1, a transcriptional suppressor that is upregulated upon Shh activation in medulloblastoma [15]. Consistent with these studies we found drastically increased Bmi1 protein (Fig. 5a) and mRNA (Fig. 5b) levels in Smo/Smo medulloblastoma when compared to normal cerebellum. Thus, Bmi1 inversely correlated with β_1_-subunit protein and mRNA levels in these tumors (Fig. 5a, b). In CGP cells, Bmi1 protein (Fig. 5c) and mRNA (Fig. 5d) levels decreased with increasing time in culture while β_1_-subunit mRNA levels increased (Fig. 5d). In SAG-treated CGP cells Bmi1 levels increased with time while SAG prevented the increase in β_1_-subunit that was observed in control cells (Fig. 5e). This inverse relationship of Bmi1 and β_1_-subunit levels in Smo/Smo tumors and in CGP cells suggested that Shh signaling-induced β_1_-subunit repression might be mediated by Bmi1. To determine whether Bmi1 represses the β_1_-subunit, we transiently expressed Bmi1 in 293 T cells and found that the β_1_-subunit protein levels decreased with increasing levels of Bmi1 (Fig. 6a). Furthermore, to test whether Bmi1 transcriptionally represses the β_1_-subunit promoter, we transiently co-transfected Bmi1 with a luciferase reporter construct under the control of the human β_1_-subunit promoter (Hβ_1_-1141-Luc) [38] in 293 T cells. Bmi1 expression significantly reduced the β_1_-subunit promoter activity to 85.7 ± 2.3 % of that of control vector transfected cells (Fig. 6b). To further confirm that Bmi1 inhibits β_1_-subunit transcription by controlling its promoter activity, we co-transfected 293 T cells with human Bmi1 shRNA and Hβ_1_-1141-Luc constructs. Silencing of Bmi1 increased β_1_-subunit promoter activity in a dose dependent manner (Fig. 6c). Together, these data show that Bmi1 represses β_1_-subunit promoter activity and down-regulates its expression.

## Discussion

In this study, we provide evidence that the β_1_-subunit of Na,K-ATPase is a target of Shh signaling *in vitro* and *in vivo* and that its expression is reduced in medulloblastoma tumors. We show that the Na,K-ATPase β_1_-subunit levels are reduced in mouse medulloblastoma from Smo/Smo mice with constitutive activation of the Shh signaling pathway. We further demonstrate that in primary cultures of CGPs from wild-type mice the β_1_-subunit levels increase with differentiation which can be prevented by activation of Shh-signaling. Downregulation of the β_1_-subunit could be mimicked by expressing the transcription factors Gli1, a well-known member of the Shh signaling cascade, and Bmi1, a polycomb protein that can be induced by Shh/Gli1 in medulloblastoma [15]. Furthermore, in human medulloblastoma cells, low β_1_-subunit levels were associated with increased cell proliferation and *in vivo* tumorigenesis. Studies from our and other groups have previously shown that Na,K-ATPase β_1_-subunit levels are low in undifferentiated carcinoma cells derived from epithelia [21, 28–31]. Using primary cultures of normal cCGPs, human medulloblastoma cell lines and a transgenic medulloblastoma mouse model, we now provide evidence that loss of β_1_-subunit expression also occurs in brain tumor cells and suggest that altered β_1_-subunit expression and function may contribute to tumor development and progression not only in carcinoma but also in tumors of neuronal origin.

It is well established that the Na,K-ATPase pump function is essential to maintain intracellular ion homeostasis and cell volume and to fulfill more tissue-specific functions like transepithelial ion and nutrient transport in epithelia or maintaining the membrane resting potential in excitable tissues. Na,K-ATPase also serves as a signaling scaffold and the β-subunit functions as a cell adhesion molecule [18–23]. The pump function of the α-subunit and the adhesion function of the β_1_-subunit are crucial for the formation and maintenance of tight junctions and the polarized phenotype of epithelial cells [21, 31, 39]. This role appears to be conserved as Na,K-ATPase function and β_1_-subunit expression are not only required for blastocyst formation in mouse embryos and tight junction formation in mammalian cells but also for the formation of septate junctions, the tight junction homolog in Drosophila, and myocardial cell junctions in zebrafish [31]. Epithelial cells exhibit a polarized distribution of proteins and lipids in the apical and basolateral plasma membrane domains. Similarly, neuronal cells are highly polarized cells with an axon and a somatodendritic domain. Many membrane proteins such as adhesion molecules, growth factor receptors, and ion channels are asymmetrically distributed into these two distinct cellular compartments that are separated by the axon initial segment (AIS). The AIS is enriched in voltage-gated ion channels, cell adhesion molecules and cytoskeletal scaffolding proteins and acts as a diffusion barrier [40]. Interestingly, recent studies showed that an increase in Na,K-ATPase α_1_-subunit preceded other proteins in the AIS and that abnormal α_1_-subunit levels resulted in altered structure and function of the AIS [41]. As cells from the central nervous system arise from neuroepithelium, it is believed that similar events in epithelial and neuronal cells shape neuronal differentiation [42]. Thus, it is possible that Na,K-ATPase function and expression are not only crucial for epithelial differentiation but also for the polarization of neuronal cells.

We have shown that loss of β_1_-subunit expression in normal epithelial cells is associated with epithelial-mesenchymal transition (EMT) [34, 35], a key step towards progression to a malignant phenotype in cancer. During EMT epithelial cells undergo a developmental switch resulting in decreased adhesion, loss of cell polarity and increased proliferation and invasiveness, a pathological process in cancer and a physiological process during normal development. During cerebellar development, neuronal precursors, and in particular CGPs, appear to undergo a switch similar to EMT [43]. In rodents, proliferating CGPs differentiate into granule neurons in the EGL and then migrate radially from the EGL into the internal granule layer (IGL) during postnatal maturation. While in the EGL, CGPs proceed through an unpolarized state with limited contact with neighboring neurons before forming neuron-glial junctions in order to move into the IGL. This process in many ways resembles EMT and the mesenchymal-epithelial transitions (MET) found in epithelial tissue formation during development. We earlier found that the β_1_-subunit expression increased during epithelial polarization (data not shown), and that the β_1_-subunit acts synergistically with the cell adhesion molecule E-cadherin to induce a polarized, epithelial phenotype [21]. We now show that the β_1_-subunit levels increase in primary cultures of CGP cells with increasing differentiation (Fig. 1c) and it is possible that in these cells the β_1_-subunit cooperates with neuronal cell adhesion molecules to induce differentiation as they undergo MET. We previously also provided evidence that the β_1_-subunit homodimerizes with the β_1_-subunit of adjacent cells [44] which was required to suppress cell proliferation in response to contact inhibition in renal epithelial cells [45]. Thus, it is also possible that in neuronal cells the β_1_-subunit plays a similar role and either may suppress proliferation in the EGL through contact inhibition or tether the CGP cells at the IGL to terminate migration. Studies are in progress to determine whether ablation of the β_1_-subunit affects the differentiation/migration of CGP cells during cerebellar development.

We have previously shown that Snail, a master regulator of EMT, binds to a non-canonical E-box in the β_1_-subunit promoter and transcriptionally suppresses the β_1_-subunit [29]. It has also been reported that activation of Shh signaling induces Snail1 in CGP cells [46]. Nevertheless, the Snail1 levels were similar in wild-type cerebella and in medulloblastoma tumors of Smo/Smo mice (Fig. 5a) suggesting that other transcription factors may play a primary role in Shh-induced downregulation of the β_1_-subunit. We previously made a similar finding in TGF-β induced EMT in human retinal pigment epithelial cells. While Snail is one of the major transcription factors induced by TGF-β signaling we did not find Snail to downregulate the β_1_-subunit in ARPE19 cells despite reduced β_1_-subunit levels in response to TGF-β treatment [35]. Rather, suppression of the β_1_-subunit was mediated by the transcription factors hypoxia-inducible factor (HIF-1α) and Smad3. While we found slightly increased Smad3 and Smad4 levels in Smo/Smo medulloblastoma as compared to cerebellum from wild-type mice (Fig. 5a), TGF-β treatment of DAOY cells did not result in reduced β_1_-subunit promoter activity despite increased levels of phosphorylated Smad3 indicating activation of the TGF-β signaling pathway in these cells (data not shown). It has previously been shown that activation of Shh or overexpression of Gli1 induced Bmi1 expression and knockdown of Bmi1 reduced medulloblastoma growth [14, 15, 47, 48]. We now found an inverse relationship between Bmi1 and β_1_-subunit levels in CGP cells and Smo/Smo medulloblastoma and that Bmi1 suppressed the promoter activity of the β_1_-subunit identifying Bmi1 as a novel transcriptional regulator of the β_1_-subunit. Analysis of two publicly available datasets, GSE3526 [49] and GSE37418 [50], revealed that compared to normal cerebellum, human medulloblastoma tumors showed decreased ATP1B1 and increased Bmi1 levels across all subgroups with a *p*-value of 4.1e-07 (ATP1B1) and 9.2e-28 (Bmi1). However, when the correlation of the ATP1B1 and Bmi1 levels was tested between the subgroups we found no significant correlation between these two genes in the individual groups (data not shown). Despite this lack of significance, these findings are consistent with previous reports analyzing the transcription expression profiles of DAOY cells with shRNA mediated knockdown of Bmi1 that found increased β_1_-subunit levels when Bmi1 was reduced [47]. While Bmi1 overexpression has been suggested as a mechanism in the pathogenesis of medulloblastoma, Bmi1 overexpression itself did not induce medulloblastoma but rather appeared to enhance tumor cell survival and proliferation [51]. Furthermore, Bmi1 was found to control medulloblastoma cell invasion, cell adhesion and motility [52]. We have previously shown that low β_1_-subunit levels are associated with decreased cell adhesion and increased invasion, motility and tumorigenesis of renal cell carcinoma cells [18, 21, 44, 45, 53]. We now show that in medulloblastoma cells decreased β_1_-subunit levels are associated with increased proliferation and tumorigenesis and suggest that at least in part Bmi1 mediates its effects on tumor progression through repression of the β_1_-subunit of Na,K-ATPase.

## Conclusions

Medulloblastoma is the most common pediatric brain tumor. It has a worse prognosis than many other pediatric cancers and understanding the molecular factors that contribute to medulloblastoma progression is a prerequisite for developing novel therapeutic approaches. It is now well accepted that Na,K-ATPase forms a macromolecular complex with cell adhesion molecules and signaling proteins and increasing evidence suggest that it is involved in the regulation of cell proliferation and migration in epithelial cells. We and others have also shown that loss of β_1_-subunit expression is associated with a poorly differentiated phenotype of carcinoma cells. We now report that reduced β_1_-subunit levels are associated with increased proliferation and invasiveness of medulloblastoma and identified Bmi1 as a novel transcriptional regulator of the β_1_-subunit while at the same time known regulators that play a major role in EMT such as Snail and TGF-β appeared to be less important. Thus, while we can learn significantly from the wealth of knowledge about signaling pathways in epithelial cancers, it appears that tumor specific regulators in medulloblastoma remain. On the other hand identification of Shh as a regulator of β_1_-subunit in cerebellar granule cells may open up the door for studies on the more recently identified roles of Shh in EMT and epithelial cancers.

## Methods

### Cell culture and transfection

Human medulloblastoma DAOY cells obtained from American Type Culture Collection (ATCC, Manassas, VA) were cultured in Minimum Essential Media (MEM) supplemented with 10 % fetal bovine serum and penicillin-streptomycin-glutamine at 37 °C and 5 % CO_2_. ONS-76 cells were kindly provided by Dr. Michael Taylor. For treatment with the Smo agonist SAG (Santa Cruz, Biotechnology, Dallas, TX), DAOY cells were serum-starved overnight and then treated with 0.1 μM SAG for indicated times.

293 T cells were cultured in Dulbecco’s modified Eagle Medium (DMEM) supplemented with 10 % fetal bovine serum and penicillin-streptomycin-glutamine at 37 °C and 5 % CO_2_. 293 T cells were transiently transfected with same amounts of control vector or human Gli1 or human Bmi1 expression constructs. Two or three days after transfection, cells were collected for further experiments. For human Gli1 and Bmi1 constructs the coding region was obtained by PCR from cDNA using the following primers: Gli forward, 5′-ACGAAGATCTATGTTCAACTCGATGACCCC-3′; Gli reverse, 5′-GAGCGTCGACTTAGGCACTAGAGTTGAGGAATTCT-3′; Bmi1 forward, 5′-ACGAAGATCTATGCATCGAACAACGAGAATCAAGATC-3′; Bmi1 reverse, 5′-GAGCGTCGAC TCAACCAGAAGAAGTTGCTGATGACC-3′. The inserts were cloned into pEGFPC1 using the BglII and SalI restriction sites. The insert was confirmed by sequencing.

For knockdown studies, the shRNA sequence (5′-GTGATGCTGCTCACCATCA-3′) that targets human Na,K-ATPase β_1_-subunit was cloned into the pSilencer 5.1 vector (Ambion, Austin, TX) as described earlier [34]. Na,K-ATPase β_1_-shRNA transfection of DAOY cells was performed using either the Amaxa Nucleofector (Lonza, Walkersville, MD) (clone 1) or Lipofectamine 2000 (Life Technologies, Grand Island, NY) (clone 2) and single clones were selected after treatment with 10 μg/ml of puromycin (Sigma-Aldrich Co. LLC, St. Louis, MO). Knockdown of the β_1_-subunit was confirmed by immunoblotting. Representative clones transfected with the scrambled sequence vector (5′-ACAGAATTGAATCATGCCGTC-3′) were selected in parallel. Two matched sets of β_1_-subunit knockdown and vector control cells were obtained from two independent transfections/selections. The shRNA sequence (5′-GACCAGACCACTACTGAAT-3′) that targets human Bmi1 was cloned into the pSIREN-DNR-DsRed-Express vector (Clontech).

### Primary culture of CGP cells

Cerebellum dissected from mice of postnatal days 4–6 were dissociated into single cells using the Papain Dissociation System kit (Worthington Biochemical Corp, Freehold, NJ). After filtering through a nylon mesh, the cell suspension was plated twice to remove astroglial cells and fibroblasts. CGPs were cultured in poly-D-lysine coated dishes in Neurobasal medium supplemented with 0.25 mM KCl and B27.

### RNA extraction and qPCR

Total RNA was extracted from cerebellum of wildtype C57BL/6 mice, medulloblastoma tumors of Smo/Smo mice and *in vitro* cultured CGP cells or DAOY cells using TRIzol reagent (Life Technologies, Carlsbad, CA). Total RNA was treated with TURBO DNA freeTM Kit (Ambion, Life Technologies). 1 μg of RNA was used to produce cDNA using the iScript cDNA Synthesis kit (Bio-Rad, Hercules, CA). Quantitative PCR analysis was performed with a SYBR Green PCR master mix (Applied Biosystems, Foster City, CA) using an ABI Prism 7900 Sequence Detection System (Applied Biosystems).

The following primer sets were used: 5′-ACCAATCTTACCATGGACACTGAA-3′ (human Na,K-β1 forward); 5′-CGGTCTTTCTCACTGTACCCAAT-3′ (human Na,K-β1 reverse); 5′-ACCCGGGGTCTCAAACTG-3' (Gli1 forward); 5′- GGCTGACAGTATAGGCAGAGC-3′ (Gli1 reverse); 5′-AATGTGGCCGAGGACTTTGATTGC-3′ (β-actin forward); and 5′-AGGATGGCAAGGGACTTCCTGTAA-3′ (β-actin reverse); 5′- TTCTGGTGCTTGTCTCACTGA-3′ (Beta2 microglobulin forward); 5′- CAGTATGTTCGGCTTCCCATTC-3′ (Beta2 microglobulin reverse); 5′- TGATCAGCATGGCCTATGGACAGA-3′ (mouse Na,K-α1 forward); 5′- TGAAAGGGCAGGAAACCGTTCTCA-3′ (mouse Na,K-α1 reverse); 5′-GGTGGCAGTTGGTTTAAGATCC-3′ (mouse Na,K-β1 forward); 5′-GGAATCTGTGTTAATCCTGG-3′ (mouse Na,K-β1 reverse); 5′-ACGTCATGTATGAAGAGGAACCT-3′ (mouse Bmi1 forward); 5′-GCAATGTCCATTAGCGTGTAGT-3′ (mouse Bmi1 reverse).

### Immunoblotting

Tissue or cell extracts were prepared either with a Triton lysis buffer (20 mM Tris–HCl, pH 7.4), 150 mM NaCl, 1 mM EDTA, 0.1 % Triton X-100, 1 mM sodium orthovanadate supplemented with protease inhibitor cocktails) or SDS lysis buffer (25 mM Tris–HCl, pH 7.4, 0.5 mM EDTA, 95 mM NaCl and 2 % SDS). Equal amounts of protein were separated by SDS-PAGE, transferred to a nitrocellulose membrane, blocked in 5 % skim milk in Tris-buffered saline with 0.1 % Tween 20 (TBST), and incubated overnight with primary antibody diluted in 5 % bovine serum albumin/TBST or 5 % milk/TBST. HRP-conjugated secondary antibodies and Enhanced Chemiluminescence Plus to visualize protein bands. Image J software was used for densitometric analysis of the immunoblots. The following antibodies were used: anti-Bmi1 (#5856), anti-Gli1 (#2643), anti-Smad4 (#9515), anti-Smad3 (#9523), anti-GAPDH (#2118), cyclin D1 (92G2) and anti-α-tubulin (#3873) from Cell Signaling Technology, anti-GFP (sc-9996) and anti-Na,K-ATPas α_1_-subunit (sc-71638) from Santa Cruz Biotechnology, anti-Na,K-ATPase β_1_-subunit (HPA012911) from Sigma, and anti-Snail (ab17732) from Abcam (Cambridge, MA).

### Luciferase assays

293 T cells were transfected with 0.1 μg of the human Na,K-ATPase β_1_-subunit promoter (Hβ1-1141-Luc) firefly luciferase reporter construct and same amounts of control vector, human Bmi1 expression construct or human Bmi1 shRNA construct. To normalize for transfection efficiency, 10 ng pRL-TK Renilla luciferase control plasmid was added to each transfection. Within the same experiment, each transfection was performed in triplicate. Two days after transfection, dual-luciferase reporter assays were performed using the dual luciferase assay kit (Promega, Madison, WI). Firefly luciferase activities were normalized to Renilla luciferase luminescence.

### Cell proliferation assay

Proliferation of Na,K-ATPase β_1_-subunit knockdown and control cells was measured by cell counting and by a BrdU incorporation assay. For cell counting, on day 0, 15,000 of both cell types were plated and the cell number in each well was determined using a hemocytometer on days 1 through 4. Final counts were averages from 3 different cell counts and values shown are averages from three independent experiments. For the BrdU incorporation assay, on day 0, 150,000 cells of both cell types were plated. On days 1 through 4, cells were labeled with 30 μM BrdU (Life Technologies) for 2 h and then harvested for flow cytometry. Cells were stained with an anti-BrdU Alexa Fluor 488 conjugate antibody (Life Technologies) and propidium iodide, then analyzed on a BD Accuri C6 flow cytometer. Values shown are averages from two independent experiments done in triplicates.

### In vivo studies

Female hairless SCID mice (Charles River Laboratories, Wilmington, MA), 5–6 weeks old, were injected subcutaneously with 7 × 10^6^ Na,K-ATPase β_1_-subunit knockdown (Sh-NaKβ-cl1) or control (ShV-cl1) DAOY cells with matrigel as described previously [54] (*n* = 12). Tumors were allowed to grow for 3 weeks and then the tumor size was measured weekly with a caliper. Tumor volumes were calculated according to the formula short diameter squared × longest diameter × 0.5. Mice that had tumors with a longest diameter of 1.7 cm were euthanized. Tumors were harvested, and tumor lysates were prepared in buffer containing 10 mM Tris–HCl, 150 mM NaCl, 1 mM EDTA, 0.1 % Triton X-100 supplemented with phosphatase and protease inhibitors. All animal studies were approved by the institutional IACUC committee.

For statistical analysis of tumor growth rates, a mixed effect model with AR(1) correlation structure was used to compare the changes in the mean volume between control (ShV-cl1) and β_1_-subunit knockdown cells (Sh-NaKβ-cl1). Variable ‘Volume’ was used the response and group, time (weeks), and interaction of group and time (Time*Group) were used as fixed factors. The id of each mouse was used as random factor to account for the heterogeneity of individual mice. The last value of the volume was carried forward for the mice sacrificed before week 12.

For survival analysis, Cox-regression for proportion model was used to compare the risk of dying (sacrificing) between two groups and a Kaplan Meier survival function was used to compare the probability of survival between two groups.

### Statistical analysis

Data are presented as mean ± SD unless otherwise indicated. Differences between means of the two groups were analyzed with the use of a two-tailed unpaired Student’s t-test. When applicable, *P* values are stated in the figure legends.

## Abbreviations

AMOG: Adhesion molecule on glia
CGP: Cerebellar granule cell precursor
EGL: External granule layer
EMT: Epithelial-mesenchymal transition
IGL: Internal granule layer
Ptch1: Patched-1
Shh: Sonic hedgehog
Smo: Smoothened
Sufu: Suppressor of fused homolog
TGF: Transforming growth factor
